# Association of smoking with serum uric acid levels, hyperuricemia, and gout based on the 7th to 9th Korea National Health and Nutrition Examination Survey: A secondary dataset analysis based on a cross-sectional study

**DOI:** 10.18332/tid/217841

**Published:** 2026-03-18

**Authors:** Sunmi Kim

**Affiliations:** 1 Department of Family Medicine, Kangwon National University College of Medicine, Chuncheon, Republic of Korea; 2 Department of Family Medicine, Kangwon National University Hospital, Chuncheon, Republic of Korea

**Keywords:** smoking, uric acid, hyperuricemia, gout, sex

## Abstract

**INTRODUCTION:**

Previous studies have reported conflicting results on the relationship of smoking with gout and hyperuricemia. This study aimed to investigate the associations of smoking with serum uric acid levels, hyperuricemia, and gout.

**METHODS:**

This study was a pooled analysis of secondary data from the 7th to 9th Korea National Health and Nutrition Examination Survey (2016–2022). We analyzed 29516 participants (12626 for gout analysis) aged ≥19 years. Smoking status (exposure) and doctor-diagnosed gout (primary outcome) were assessed through self-reported questionnaires, while serum uric acid levels (secondary outcome) were measured from blood samples. Multiple linear and logistic regression analyses were performed to assess the associations, adjusting for potential confounders.

**RESULTS:**

In females, current smokers had significantly higher serum uric acid levels than never smokers (adjusted mean difference=0.16 mg/dL; 95% CI: 0.08–0.24; p<0.001). Current smoking in females was also significantly associated with increased odds of both hyperuricemia (adjusted odds ratio, AOR=1.49; 95% CI: 1.11–2.00; p=0.008) and self-reported doctor-diagnosed gout (AOR=22.07; 95% CI: 6.66–73.09; p<0.001) compared with never smoking. In contrast, no significant associations were observed in males; the adjusted mean difference in serum uric acid levels between current and never smokers was -0.01 mg/dL (95% CI: -0.08–0.05; p=0.668), and the AOR for gout was 1.00 (95% CI: 0.62–1.59; p=0.984).

**CONCLUSIONS:**

The results suggest that smoking is associated with elevated serum uric acid levels and an increased prevalence of hyperuricemia and gout in women but not in men.

## INTRODUCTION

Gout is the most common form of inflammatory arthritis and results from the deposition of monosodium urate crystals in joints and surrounding tissues. The crystals form due to supersaturation of uric acid in the tissues, which results from hyperuricemia^1^.

Gout and hyperuricemia are associated with various chronic diseases such as hypertension, diabetes, coronary artery disease, and chronic kidney disease, all of which share smoking as a common risk factor^2^. On the other hand, gout and hyperuricemia are closely related to obesity, but smoking shows an inverse relationship to obesity^2^. These conflicting associations with the various factors can make it difficult to determine the definite relationship between smoking and gout.

Previous studies have reported conflicting results on the relationship of smoking with gout and hyperuricemia, depending on the population and methods used in the study. Some studies showed that smoking is associated with elevated serum uric acid levels and an increased risk of gout^3-6^. In contrast, other studies reported that smoking reduces serum uric acid levels and has a protective effect against the risk of gout^7-10^. Some studies failed to prove the association of smoking with gout and hyperuricemia^11^. In addition, sex-specific differences in the relationship were also noted in some of the studies^3,5-9^. Due to the conflicting evidence, the effect of smoking on hyperuricemia and gout is still controversial. Thus, this study aimed to investigate whether smoking is associated with higher serum uric acid levels, hyperuricemia, and gout.

## METHODS

### Study population

**Figure 1:**
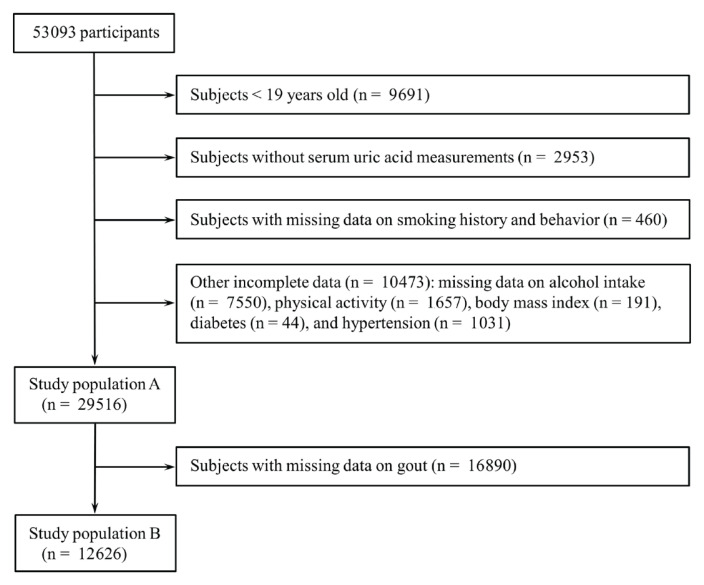
Flowchart of participant selection from the 7th to 9th Korea National Health and Nutrition Examination Survey (2016–2022) for this pooled cross-sectional study

This study is a secondary analysis of pooled cross-sectional data from the 2016–2022 Korea National Health and Nutrition Examination Survey (KNHANES). The KNHANES is a nationally representative cross-sectional survey that includes a health interview, physical examination, and laboratory tests^12^. The KNHANES measured serum uric acid levels in 2016–2022, and the eighth KNHANES (2019–2021) collected data on doctor-diagnosed gout. Hence, this study examined data from 53093 participants collected between 2016 and 2022 to assess the association of smoking with serum uric acid levels and hyperuricemia. Participants younger than 19 years were excluded because data on smoking were not collected from children, and the questionnaire items on smoking in adolescents were different from those in adults. Additional exclusions were applied to participants without serum uric acid measurements, those with missing data on smoking history and behavior, and those with incomplete data on covariates. After these exclusions, a total of 29516 subjects were eligible for the evaluation of the association of smoking with serum uric acid levels and hyperuricemia (study population A). Among them, data on doctor-diagnosed gout were available in 12626 participants, who composed the study population B to determine the association between smoking and gout (Figure 1).

### Definitions of variables

The primary outcome was doctor-diagnosed gout, and the secondary outcomes were mean serum uric acid levels and hyperuricemia. The doctor-diagnosed gout was assessed using the questionnaire item: ‘Have you been diagnosed with gout by a doctor?’. Serum uric acid levels were measured using uricase-based enzymatic colorimetric assays on automated analyzers. Hyperuricemia was defined as a serum uric acid level higher than 7 mg/dL in men and 6 mg/dL in women^13^.

Smoking status was assessed using a standardized questionnaire and classified as never smoker, ex-smoker, and current smoker. Participants who smoked fewer than 100 cigarettes during their lifetime were defined as never smokers; those who had smoked in the past but did not currently smoke were defined as ex-smokers; those who currently smoked were defined as current smokers^14^. Among current smokers, exposure to smoking was assessed based on self-reported smoking duration, smoking intensity, and cumulative dose of smoking. Smoking intensity was defined as the average number of cigarettes smoked per day, and cumulative dose was calculated as the product of smoking intensity and smoking duration. Based on the distribution among current smokers, the median or a value close to the median was used to categorize the smoking exposure variables. Specifically, smoking duration was categorized as <20 years and ≥20 years (median=23); smoking intensity as <10 cigarettes per day and ≥10 cigarettes per day (median=10); and cumulative smoking dose as <10 pack-years and ≥10 pack-years (median=12.0)^15^.

Age was obtained through a questionnaire and classified as: <30, 30 to <40, 40 to <50, 50 to <60, 60 to <70, and ≥70 years. Physical activity was assessed using the Global Physical Activity Questionnaire (GPAQ), and GPAQ scores were categorized into low, medium, and high^16^. Alcohol intake was assessed via questionnaire and calculated as the average daily alcohol consumption by multiplying drinking frequency by the amount of alcohol consumed per occasion^17^. Based on the calculated amount, alcohol intake was classified as: 0, <16, 16–40, and >40 g/day^18^.

Body mass index was calculated as weight (kg) divided by height (m) squared (kg/m^2^), with height and weight measured by trained personnel during the health examination, and categorized according to the guidelines of the Korean Society for the Study of Obesity: <18.5, 18.5 to <23, 23 to <25, 25 to <30, 30 to <35, and ≥35^19^. Estimated glomerular filtration rates were calculated in mL/min/1.73 m^2^ using the Modification of Diet and Renal Disease formula based on measured serum creatinine levels, age, and sex^20^, and categorized as: <60, 60 to <90, and ≥90^21^. Diabetes was defined based on measured fasting plasma glucose (≥126 mg/dL), measured glycosylated hemoglobin (HbA1c ≥6.5%), self-reported prior diagnosis of diabetes, or current use of antidiabetic medications^22^. Hypertension was defined as measured systolic blood pressure ≥140 mmHg, diastolic blood pressure ≥90 mmHg, or use of anti-hypertensive medications^23^. Dyslipidemia was defined as having one or more of the following based on laboratory measurements or questionnaire data: total cholesterol ≥240 mg/dL, triglyceride ≥150 mg/dL, low-density lipoprotein cholesterol ≥160 mg/dL, high-density lipoprotein cholesterol level of <40 mg/dL for men and <50 mg/dL for women, previous doctor-diagnosed dyslipidemia, or current use of lipid-lowering drugs^24^.

### Statistical analysis

As the KNHANES sampling plan followed a multi-stage clustered probability design, statistical analyses used sample weights to account for the complex sampling design and yield nationally representative estimates^12^. Statistical analyses were conducted using the statistical software package R version 4.2.3 (The R Foundation for Statistical Computing, Vienna, Austria), and a two-sided p<0.05 was considered statistically significant.

The proportions and standard errors of demographic, health-behavioral, and clinical variables were estimated by hyperuricemia and gout status. The differences in the variables according to hyperuricemia and gout were assessed using the Pearson χ^2^ test with Rao-Scott adjustment.

Multiple linear regression analyses were performed to estimate the mean serum uric acid levels according to the status, duration, intensity, and cumulative dose of smoking in the study population A. The multiple linear regression models included covariates such as sex, age, physical activity, alcohol intake, body mass index, estimated glomerular filtration rate, diabetes, hypertension, and dyslipidemia, and included the interaction between the smoking factors and sex. When the interaction was statistically significant, sex-stratified multiple linear regression analyses were conducted separately in females and males.

To estimate adjusted odds ratios (AORs) and 95% confidence intervals (CIs) for hyperuricemia by smoking status, duration, intensity, and cumulative dose in study population A, multiple logistic regression analyses were conducted. The multiple logistic regression models included the same covariates as mentioned above, along with the interaction between the smoking factors and sex. When the interaction was statistically significant, sex-stratified multiple logistic regression analyses were conducted separately in females and males.

To estimate AORs and 95% CIs for gout by status, duration, intensity, and cumulative smoking dose in study population B, multiple logistic regression analyses were performed in the same manner as in the hyperuricemia analyses described above. To address the potential bias from sparse data (a small number of female smokers with gout), we performed a sensitivity analysis using Firth’s penalized likelihood logistic regression. This sensitivity analysis did not incorporate the complex sampling weights due to methodological limitations.

## RESULTS

**Figure 2:**
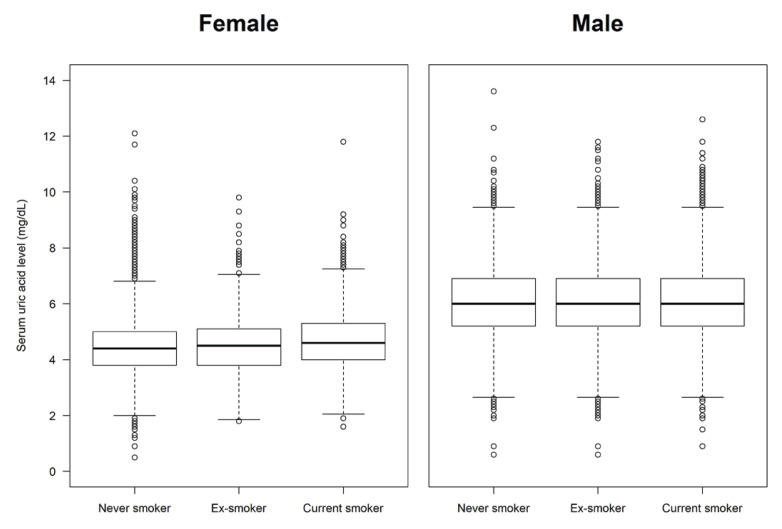
The distributions of serum uric acid levels according to smoking status in female and male participants: A pooled cross-sectional analysis of the 7th to 9th Korea National Health and Nutrition Examination Survey (2016–2022) (N=29516)

A total of 29516 participants were included in the analysis for hyperuricemia (study population A). The mean age of the participants was 46.0 years (SD=16.2). Males accounted for 51.4% of the population, and females accounted for 48.6%. Regarding smoking status, 58.5% were never smokers, 20.4% were ex-smokers, and 21.1% were current smokers. The overall prevalence of hyperuricemia was 13.9%. In the analysis for gout (study population B, n=12626), the prevalence of doctor-diagnosed gout was 2.0%. The demographic, health behavioral, and clinical characteristics of the study population are summarized in Table 1. The smoking status was significantly different between participants with and without hyperuricemia (p<0.001), and was also different between those with and without gout (p<0.001). The box plots of serum uric acid levels by smoking status showed that levels tended to increase in the order never smoker, ex-smoker, and current smoker in females, but not in males (Figure 2).

**Table 1:** General characteristics of study participants according to hyperuricemia and gout status: A pooled cross-sectional analysis of the 7th to 9th Korea National Health and Nutrition Examination Survey (2016–2022) (Study population A, N=29516; Study population B, N=12626)

*Characteristics*	*Study population A*				*Study population B*			
		*Hyperuricemia*				*Gout*		
	*Total*	*No*	*Yes*	*p*	*Total*	*No*	*Yes*	*p*
**Total**								
N	29516	25784	3732		12626	12391	235	
Percent	100	86.1 ± 0.3	13.9 ± 0.3		100	98.0 ± 0.1	2.0 ± 0.1	
**Smoking status**				<0.001				<0.001
Never smoker	58.5 ± 0.4	61.1 ± 0.4	42.9 ± 0.9		58.0 ± 0.5	58.7 ± 0.5	21.5 ± 2.9	
Ex-smoker	20.4 ± 0.3	19.2 ± 0.3	27.6 ± 0.8		21.2 ± 0.4	20.7 ± 0.4	46.5 ± 3.7	
Current smoker	21.1 ± 0.3	19.7 ± 0.3	29.5 ± 0.9		20.9 ± 0.5	20.6 ± 0.5	32.0 ± 3.6	
**Sex**				<0.001				<0.001
Female	48.6 ± 0.3	53.0 ± 0.3	22.0 ± 0.7		47.9 ± 0.4	48.7 ± 0.4	8.6 ± 1.8	
Male	51.4 ± 0.3	47.0 ± 0.3	78.0 ± 0.7		52.1 ± 0.4	51.3 ± 0.4	91.4 ± 1.8	
**Age** (year)				<0.001				<0.001
<30	19.8 ± 0.4	19.0 ± 0.4	24.5 ± 1.0		20.4 ± 0.6	20.7 ± 0.6	4.6 ± 1.7	
30 to <40	18.2 ± 0.4	17.3 ± 0.4	23.7 ± 0.9		18.2 ± 0.6	18.2 ± 0.6	15.8 ± 3.1	
40 to <50	20.5 ± 0.4	20.7 ± 0.4	19.6 ± 0.8		20.0 ± 0.5	19.8 ± 0.5	26.2 ± 3.4	
50 to <60	19.3 ± 0.3	20.1 ± 0.3	14.0 ± 0.6		19.2 ± 0.5	19.2 ± 0.5	21.6 ± 3.2	
60 to <70	13.1 ± 0.3	13.7 ± 0.3	9.4 ± 0.5		13.3 ± 0.4	13.1 ± 0.4	22.7 ± 2.9	
≥70	9.2 ± 0.2	9.2 ± 0.2	8.7 ± 0.4		9.0 ± 0.4	8.9 ± 0.4	9.1 ± 1.6	
**Physical activity**				<0.001				0.780
Low	58.9 ± 0.4	59.5 ± 0.4	54.8 ± 0.9		60.3 ± 0.6	60.3 ± 0.6	60.9 ± 3.6	
Moderate	30.6 ± 0.4	30.6 ± 0.4	30.6 ± 0.9		29.6 ± 0.5	29.6 ± 0.5	30.6 ± 3.6	
High	10.5 ± 0.2	9.9 ± 0.2	14.6 ± 0.7		10.1 ± 0.4	10.1 ± 0.4	8.6 ± 2.0	
**Alcohol** (g/day)				<0.001				<0.001
0	9.7 ± 0.2	10.2 ± 0.2	6.5 ± 0.4		9.6 ± 0.3	9.8 ± 0.3	3.8 ± 1.1	
<16	70.0 ± 0.3	71.2 ± 0.3	62.6 ± 1.0		70.0 ± 0.5	70.4 ± 0.5	51.0 ± 3.8	
16–40	12.5 ± 0.2	11.8 ± 0.2	17.0 ± 0.7		12.0 ± 0.3	11.8 ± 0.3	23.1 ± 3.1	
>40	7.8 ± 0.2	6.8 ± 0.2	13.9 ± 0.7		8.3 ± 0.3	8.1 ± 0.3	22.1 ± 3.2	
**Body mass index** (kg/m^2^)				<0.001				<0.001
<18.5	4.1 ± 0.1	4.6 ± 0.2	1.1 ± 0.2		4.1 ± 0.2	4.1 ± 0.2	2.7 ± 1.3	
18.5 to <23	37.2 ± 0.3	40.3 ± 0.4	18.2 ± 0.7		36.1 ± 0.5	36.6 ± 0.5	15.6 ± 2.7	
23 to <25	22.6 ± 0.3	22.8 ± 0.3	21.4 ± 0.8		22.5 ± 0.4	22.4 ± 0.4	24.0 ± 3.2	
25 to < 30	29.9 ± 0.3	27.6 ± 0.3	43.5 ± 0.9		30.5 ± 0.5	30.3 ± 0.5	41.3 ± 3.7	
30 to < 35	5.2 ± 0.2	4.1 ± 0.2	12.2 ± 0.6		5.6 ± 0.3	5.5 ± 0.3	12.8 ± 2.6	
≥35	1.0 ± 0.1	0.6 ± 0.1	3.6 ± 0.4		1.2 ± 0.1	1.2 ± 0.1	3.5 ± 1.6	
**Estimated GFR^a^**				<0.001				<0.001
≥90	67.9 ± 0.4	69.8 ± 0.4	55.7 ± 1.0		69.0 ± 0.6	69.5 ± 0.6	47.7 ± 3.8	
60 to <90	30.5 ± 0.4	29.1 ± 0.4	39.0 ± 1.0		29.3 ± 0.5	29.1 ± 0.6	43.4 ± 3.7	
<60	1.7 ± 0.1	1.1 ± 0.1	5.3 ± 0.3		1.6 ± 0.1	1.5 ± 0.1	8.9 ± 2.0	
**Diabetes**				0.247				0.018
No	90.8 ± 0.2	90.9 ± 0.2	90.3 ± 0.5		90.3 ± 0.3	90.4 ± 0.3	85.3 ± 2.6	
Yes	9.2 ± 0.2	9.1 ± 0.2	9.7 ± 0.5		9.7 ± 0.3	9.6 ± 0.3	14.7 ± 2.6	
**Hypertension**				<0.001				<0.001
No	72.8 ± 0.4	74.0 ± 0.4	65.8 ± 0.9		73.1 ± 0.5	73.7 ± 0.5	42.0 ± 3.8	
Yes	27.2 ± 0.4	26.0 ± 0.4	34.2 ± 0.9		26.9 ± 0.5	26.3 ± 0.5	58.0 ± 3.8	
**Dyslipidemia**				<0.001				<0.001
No	46.5 ± 0.4	49.1 ± 0.4	30.9 ± 0.9		46.5 ± 0.6	46.9 ± 0.6	25.8 ± 3.3	
Yes	53.5 ± 0.4	50.9 ± 0.4	69.1 ± 0.9		53.5 ± 0.6	53.1 ± 0.6	74.2 ± 3.3	

Data are presented as weighted percentages ± standard errors. P-values were obtained by Pearson chi-squared test with Rao-Scott adjustment. a Estimated GFR are presented in units of mL/min/1.73 m^2^. GFR: glomerular filtration rate.

**Figure 3:**
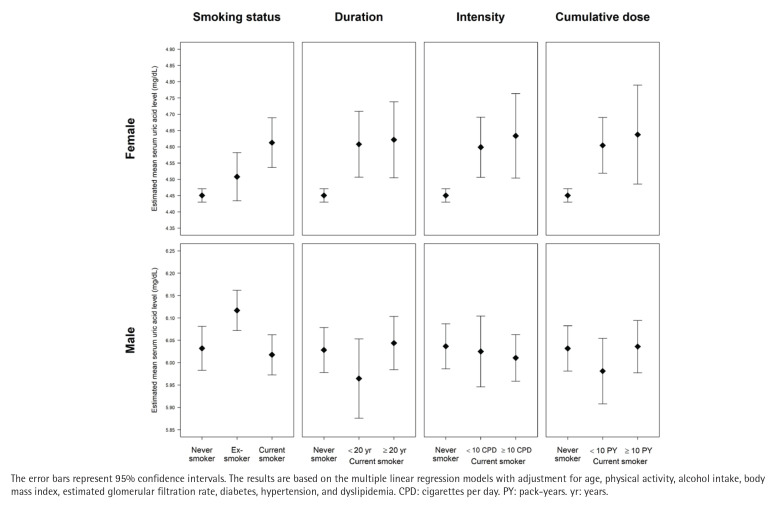
Estimated mean serum uric acid levels according to the status, duration, intensity, and cumulative dose of smoking in female and male participants: A pooled cross-sectional analysis of the 7th to 9th Korea National Health and Nutrition Examination Survey (2016–2022) (N=29516)

Significant interactions were observed between sex and smoking behaviors regarding serum uric acid levels. Specifically, the interactions of sex with smoking status, smoking duration, smoking intensity, and cumulative dose were all statistically significant (all p for interaction <0.01). Therefore, the multiple linear regression analyses were performed separately for females and males. In females, the adjusted mean serum uric acid level in current smokers was significantly higher than that in never smokers, with an adjusted mean difference of 0.16 mg/dL (95% CI: 0.08–0.24; p<0.001). Furthermore, adjusted mean serum uric acid levels increased significantly with increasing smoking duration, smoking intensity, and cumulative dose in females (all p for trend <0.001). On the other hand, in males, the adjusted mean serum uric acid levels showed no significant difference between current smokers and never smokers (adjusted mean difference= -0.01 mg/dL; 95% CI: -0.08–0.05; p=0.668), and showed no significant differences depending on smoking duration, smoking intensity, and cumulative dose of smoking (Figure 3 and Table 2).

**Table 2:** Adjusted mean differences in serum uric acid levels in female and male participants: A pooled cross-sectional analysis of the 7th to 9th Korea National Health and Nutrition Examination Survey (2016–2022) (Study population A, N=29516)

*Smoking behaviors*	*Female*				*Male*			
	*Mean difference (95% CI)*				*Mean difference (95% CI)*			
	*Crude*	*Adjusted*	*p*	*p for trend*	*Crude*	*Adjusted*	*p*	*p for trend*
**Never smoker:** reference for all comparisons	Ref.	Ref.			Ref.	Ref.		
**Smoking status**								
Ex-smoker	0.12 (0.03–0.20)	0.06 (-0.02–0.13)	0.145		-0.06 (-0.13–0.00)	0.08 (0.02–0.15)	0.010	
Current smoker	0.28 (0.20–0.36)	0.16 (0.08–0.24)	<0.001		-0.03 (-0.10–0.04)	-0.01 (-0.08–0.05)	0.668	
**Years of smoking in current smokers**				<0.001				0.918
<20	0.33 (0.22–0.44)	0.16 (0.05–0.26)	0.003		0.26 (0.17–0.36)	-0.06 (-0.16–0.03)	0.202	
≥20	0.21 (0.09–0.33)	0.17 (0.05–0.29)	0.004		-0.20 (-0.28 – -0.13)	0.02 (-0.06–0.10)	0.703	
**Intensity of smoking in current smokers** (CPD)				<0.001				0.486
<10	0.26 (0.16–0.36)	0.15 (0.05–0.24)	0.002		0.03 (-0.08–0.13)	-0.01 (-0.11–0.08)	0.809	
≥10	0.31 (0.17–0.45)	0.18 (0.05–0.31)	0.006		-0.05 (-0.12–0.02)	-0.03 (-0.10–0.05)	0.485	
**Cumulative dose of smoking in current smokers** (PY)				<0.001				0.908
<10	0.28 (0.19–0.37)	0.15 (0.07–0.24)	0.001		0.17 (0.08–0.25)	-0.05 (-0.14–0.04)	0.250	
≥10	0.28 (0.12–0.44)	0.19 (0.03–0.34)	0.016		-0.16 (-0.23 – -0.08)	0.00 (-0.08–0.08)	0.919	

The adjusted mean differences and p-values were calculated using multiple linear regression analyses with adjustment for age, physical activity, alcohol intake, body mass index, estimated glomerular filtration rate, diabetes, hypertension, and dyslipidemia. CPD: cigarettes per day. PY: pack-years.

Significant interactions were observed between sex and all smoking behaviors regarding hyperuricemia (all p for interaction <0.01). Thus, the multiple logistic regression analyses for hyperuricemia were conducted separately in females and males. In females, the odds of hyperuricemia in current smokers were significantly higher than those in never smokers with adjustment for potential confounding factors (AOR=1.49; 95% CI: 1.11–2.00; p=0.008). Additionally, when adjusted for confounding factors, the odds of hyperuricemia increased as smoking duration, smoking intensity, and cumulative dose increased in females (all p for trend ≤0.004). However, in males, the adjusted odds of hyperuricemia were not significantly different between current smokers and never smokers (AOR=0.90; 95% CI: 0.78–1.03; p=0.118), and showed no significant differences depending on smoking duration and smoking intensity. In addition, the adjusted odds of hyperuricemia in males did not change proportionally with the cumulative dose of smoking (Table 3).

**Table 3:** Odds ratios for hyperuricemia in female and male participants: A pooled cross-sectional analysis of the 7th to 9th Korea National Health and Nutrition Examination Survey (2016–2022) (Study population A, N=29516)

*Smoking behaviors*	*Female*				*Male*			
	*OR (95% CI)*	*AOR (95% CI)*	*p*	*p for trend*	*OR (95% CI)*	*AOR (95% CI)*	*p*	*p for trend*
**Never smoker:** reference for all comparisons	Ref.	Ref.			Ref.	Ref.		
**Smoking status**								
Ex-smoker	1.13 (0.82–1.56)	0.97 (0.69–1.38)	0.884		0.91 (0.81–1.03)	1.05 (0.92–1.20)	0.500	
Current smoker	1.81 (1.41–2.32)	1.49 (1.11–2.00)	0.008		0.94 (0.83–1.06)	0.90 (0.78–1.03)	0.118	
**Years of smoking in current smokers**				<0.001				0.315
<20	1.69 (1.19–2.39)	1.21 (0.80–1.83)	0.374		1.28 (1.09–1.50)	0.82 (0.68–1.00)	0.053	
≥20	2.00 (1.44–2.78)	2.00 (1.35–2.95)	0.001		0.76 (0.67–0.88)	0.97 (0.82–1.16)	0.772	
**Intensity of smoking in current smokers** (CPD)				0.004				0.130
<10	1.61 (1.14–2.28)	1.30 (0.87–1.95)	0.201		0.98 (0.81–1.18)	0.89 (0.73–1.09)	0.274	
≥10	2.09 (1.47–2.96)	1.74 (1.16–2.60)	0.007		0.93 (0.82–1.05)	0.89 (0.76–1.04)	0.130	
**Cumulative dose of smoking in current smokers** (PY)				0.003				0.409
<10	1.67 (1.23–2.26)	1.32 (0.93–1.87)	0.123		1.12 (0.96–1.32)	0.83 (0.69–0.99)	0.040	
≥10	2.20 (1.45–3.33)	1.95 (1.20–3.20)	0.008		0.83 (0.73–0.96)	0.96 (0.81–1.14)	0.672	

AOR: adjusted odds ratio. The AORs and p-values were calculated using multiple logistic regression analyses with adjustment for age, physical activity, alcohol intake, body mass index, estimated glomerular filtration rate, diabetes, hypertension, and dyslipidemia. CPD: cigarettes per day. PY: pack-years.

Similarly, the interactions between sex and smoking behaviors for gout were all statistically significant (all p for interaction <0.01). Therefore, multiple logistic regression analyses for gout were conducted separately for females and males. Among females, the odds of gout in current smokers were significantly higher than in never smokers after adjustment for potential confounding factors (AOR=22.07; 95% CI: 6.66–73.09; p<0.001). Furthermore, when adjusted for confounding factors, the odds of gout increased as smoking intensity and cumulative dose increased in females (p for trend <0.001 for both) (Table 4). In a sensitivity analysis using Firth’s bias-reduced logistic regression to account for sparse data, the associations remained statistically significant, although the magnitudes of the AORs were attenuated (e.g. AOR for current vs never smokers 13.55; 95% CI: 4.90–35.93; p<0.001) (Table 5). However, in males, the adjusted odds of gout were not significantly different between current smokers and never smokers (AOR=1.00; 95% CI: 0.62–1.59; p=0.984), and showed no significant differences depending on smoking duration, smoking intensity, and cumulative dose of smoking (Table 4).

**Table 4:** Odds ratios for gout in female and male participants: A pooled cross-sectional analysis of the 7th to 9th Korea National Health and Nutrition Examination Survey (2016–2022) (Study population B, N=12626)

*Smoking behaviors*	*Female*				*Male*			
	*OR (95% CI)*	*AOR (95% CI)*	*p*	*p for trend*	*OR (95% CI)*	*AOR (95% CI)*	*p*	*p for trend*
**Never smoker:** reference for all comparisons	Ref.	Ref.			Ref.	Ref.		
**Smoking status**								
Ex-smoker	2.04 (0.45–9.25)	2.56 (0.53–12.35)	0.240		2.40 (1.58–3.67)	1.35 (0.87–2.07)	0.178	
Current smoker	8.39 (3.06–23.00)	22.07 (6.66–73.09)	<0.001		1.56 (0.98–2.48)	1.00 (0.62–1.59)	0.984	
**Years of smoking in current smokers**				<0.001				0.975
<20	8.50 (2.24–32.17)	31.93 (8.18–124.71)	<0.001		0.94 (0.45–1.94)	0.94 (0.37–2.39)	0.889	
≥20	8.22 (2.50–27.08)	14.45 (3.42–61.01)	<0.001		1.95 (1.20–3.18)	1.00 (0.60–1.67)	0.997	
**Intensity of smoking in current smokers** (CPD)				<0.001				0.888
<10	6.63 (2.10–20.91)	18.24 (4.14–80.42)	<0.001		1.14 (0.56–2.31)	0.84 (0.41–1.75)	0.649	
≥10	10.94 (2.52–47.45)	23.69 (5.22–107.58)	<0.001		1.72 (1.06–2.80)	1.03 (0.60–1.77)	0.925	
**Cumulative dose of smoking in current smokers** (PY)				<0.001				0.986
<10	5.86 (1.99–17.25)	18.99 (4.60–78.39)	<0.001		1.04 (0.53–2.02)	0.95 (0.45–2.00)	0.890	
≥10	15.51 (3.17–75.78)	23.12 (4.05–131.81)	<0.001		1.92 (1.17–3.16)	0.99 (0.58–1.70)	0.983	

AOR: adjusted odds ratio. The AORs and p-values were calculated using multiple logistic regression analyses with adjustment for age, physical activity, alcohol intake, body mass index, estimated glomerular filtration rate, diabetes, hypertension, and dyslipidemia. CPD: cigarettes per day. PY: pack-years.

**Table 5:** Sensitivity analysis using Firth’s penalized likelihood logistic regression for the association between smoking and gout in female participants: A pooled cross-sectional analysis of the 7th to 9th Korea National Health and Nutrition Examination Survey (2016–2022) (Study population B, N=12626)

*Smoking behaviors*	*OR (95% CI)*	*AOR (95% CI)*	*p*	*p for trend*
**Never smoker:** reference for all comparisons	Ref.	Ref.		
**Smoking status**				
Ex-smoker	2.20 (0.44–6.93)	2.67 (0.47–10.15)	0.237	
Current smoker	6.89 (2.90–15.05)	13.55 (4.90–35.93)	<0.001	
**Years of smoking in current smokers**				<0.001
<20	6.66 (2.07–17.20)	18.14 (4.54–63.51)	<0.001	
≥20	8.05 (2.49–20.84)	11.03 (3.00–34.29)	0.001	
**Intensity of smoking in current smokers** (CPD)				<0.001
<10	6.41 (1.99–16.56)	11.41 (3.10–36.04)	0.001	
≥10	8.44 (2.61–21.86)	16.59 (4.55–52.31)	<0.001	
**Cumulative dose of smoking in current smokers** (PY)				<0.001
<10	6.49 (2.25–15.79)	13.96 (4.15–42.43)	<0.001	
≥10	9.03 (2.37–25.48)	13.11 (3.11–44.24)	0.001	

This sensitivity analysis did not incorporate the complex sampling weights. AOR: adjusted odds ratio. The AORs and p-values were calculated using multiple Firth’s penalized likelihood logistic regression analyses with adjustment for age, physical activity, alcohol intake, body mass index, estimated glomerular filtration rate, diabetes, hypertension, and dyslipidemia. CPD: cigarettes per day. PY: pack-years.

## DISCUSSION

The findings from this study show clear associations of smoking with serum uric acid levels, hyperuricemia, and gout in females, but not in males. In females, current smokers had higher serum uric acid levels than never smokers, and both hyperuricemia and gout were significantly more frequent in current smokers than in never smokers. In addition, this study revealed that as smoking intensity and cumulative dose of smoking increased in females, serum uric acid levels and frequencies of hyperuricemia and gout also increased. On the other hand, no such associations were observed in males. These results are consistent with previous studies^3,5,6^.

However, these results are contradictory to some previous studies that reported inverse associations of smoking with hyperuricemia and gout, particularly in males, but no associations in females^7-9^. An experimental study also reported that plasma uric acid levels significantly decreased 5 minutes after smoking a single cigarette^25^. The reason for these conflicting results is unclear, but it might be attributed to the complex effects of smoking on the production and elimination of uric acid. Cigarette smoking induces oxidative stress through reactive oxygen species and free radicals^2^. Uric acid is the most abundant water-soluble antioxidant in serum, and acts as a scavenger of free radicals and reactive oxygen species^2^. Therefore, it can be speculated that the oxidative effect of smoking may lead to a depletion of uric acid in serum^2^. It has also been argued that cyanide in cigarette smoke may inhibit xanthine oxidase, thereby reducing uric acid production to lower serum uric acid levels^2^. Researchers supporting the inverse association between smoking and hyperuricemia suggest that this mechanism may be responsible for their results^2,9^.

However, the effect of smoking on serum uric acid levels does not appear to be so straightforward. Given the role of uric acid as a major antioxidant^2^, prolonged oxidative stress may lead to an increase in serum uric acid levels for self-protection against oxidative damage. This is supported by experiments using Drosophila melanogaster larvae. Uric acid plays a role in protecting the larvae from the toxicity of cigarette smoke^26^, and continuous exposure to cigarette smoke for 6 hours has been reported to increase uric acid levels in D. melanogaster larvae^27^.

Furthermore, unlike the short-term effects of smoking, long-term smoking may have different effects on serum uric acid levels. Repeated exposure to cigarette smoke for 2 months increased serum uric acid levels in rats^28^. Cigarette smoking is known to reduce renal blood flow and increase plasma endothelin-1 levels, thereby impairing kidney function^28^. Since two-thirds of uric acid excretion occurs through the kidneys^2^, smoking may increase serum uric acid levels by reducing the excretion of uric acid^28^.

In this study, cigarette smoking was associated with serum uric acid levels, hyperuricemia, and gout only in females, indicating that sex acts as an effect modifier. This effect modification suggests that the impact of smoking differs by sex, potentially due to underlying molecular or physiological mechanisms. One plausible mechanism is the antiestrogenic effect of smoking^29^. Estrogen is known to decrease serum uric acid levels by promoting uric acid excretion through reduction of post-secretory tubular reabsorption of uric acid^30^, and by decreasing uric acid production through inhibition of the activity of xanthine oxidase^31^. Cigarette smoking may prevent these actions of estrogen and thus increase serum uric acid levels, especially in females.

This study analyzed serum uric acid levels, hyperuricemia, and gout, and all of them were consistently associated with smoking status and exposure in females. This consistency made the study’s findings more reliable. In addition, the results were obtained with adjustment for a wide variety of potential confounding factors, including age, physical activity, alcohol intake, body mass index, estimated glomerular filtration rate, diabetes, hypertension, and dyslipidemia. Because this study is based on nationally representative data, its results can be generalized to the general adult population in Korea.

### Limitations

However, this study also has several limitations. First, because the KNHANES is a cross-sectional survey, this study can confirm the association but cannot establish a causal relationship. Specifically, the possibility of reverse causality cannot be excluded; for instance, participants diagnosed with gout or hyperuricemia might have altered their smoking habits due to their health condition. Second, a substantial number of participants were excluded from the analysis due to missing data. If the exclusion of these participants was related to both smoking habits and the outcomes, it may have introduced selection bias. Third, the diagnosis of gout relied solely on the participant’s self-report. This reliance on self-reported data may introduce information bias, specifically misclassification bias, due to inaccuracies in recall of diagnoses. Fourth, our definition of smoking exposure was limited to conventional combustible cigarettes and did not account for the use of newer tobacco products, such as electronic cigarettes (e-cigarettes) or heated tobacco products. Since the survey questions regarding these products changed frequently during the study period (2016–2022), it was not methodologically feasible to construct a consistent variable for the entire pooled dataset. Consequently, we could not evaluate the potential differential impacts of e-cigarettes or the dual use of conventional and e-cigarettes on serum uric acid levels. Fifth, despite adjusting for multiple covariates, the possibility of residual confounding remains. Specifically, information on dietary purine intake and diuretic medication use was not available in the KNHANES dataset. Additionally, coffee consumption could not be included in the analysis because the survey questions regarding coffee intake varied across the study years, preventing data pooling. These unmeasured factors might have influenced the observed associations. Sixth, the AORs for gout in females were notably high with wide CIs. This statistical instability is likely attributable to the low prevalence of gout in females, which results in few cases and potential sparse-data bias. To address this issue, we performed a sensitivity analysis using Firth’s penalized likelihood logistic regression, which corrects for sparse data bias. The results showed attenuated but still substantial ORs, and the association remained statistically significant. This suggests that while the magnitude of the weighted estimate in the main analysis should be interpreted with caution, the strong association between smoking and gout in women is robust and not merely a statistical artifact. This is further supported by consistent results from the linear regression analysis of serum uric acid levels. Seventh, because KNHANES produces data representative of Koreans, it is not appropriate to directly extrapolate this study’s results to people in other countries.

## CONCLUSIONS

This study provides epidemiological evidence of associations of smoking with serum uric acid levels, hyperuricemia, and gout in women but not in men. Additional studies, particularly prospective cohort studies, are needed to verify the causality, determine the generalizability to other countries, and elucidate the underlying mechanism.

## Data Availability

The data supporting this research are available from the following source: https://knhanes.kdca.go.kr/knhanes/main.do
